# Population Pharmacokinetic and Pharmacodynamic Analysis of Dalbavancin for Long-Term Treatment of Subacute and/or Chronic Infectious Diseases: The Major Role of Therapeutic Drug Monitoring

**DOI:** 10.3390/antibiotics11080996

**Published:** 2022-07-24

**Authors:** Pier Giorgio Cojutti, Sara Tedeschi, Milo Gatti, Eleonora Zamparini, Marianna Meschiari, Paola Della Siega, Maria Mazzitelli, Laura Soavi, Raffaella Binazzi, Elke Maria Erne, Marco Rizzi, Anna Maria Cattelan, Carlo Tascini, Cristina Mussini, Pierluigi Viale, Federico Pea

**Affiliations:** 1 Clinical Pharmacology Unit, Department for Integrated Infectious Risk Management, IRCCS Azienda Ospedaliero-Universitaria di Bologna, 40138 Bologna, Italy; piergiorgio.cojutti@aosp.bo.it (P.G.C.); milo.gatti2@unibo.it (M.G.); 2 Infectious Diseases Unit, Department for Integrated Infectious Risk Management, IRCCS Azienda Ospedaliero-Universitaria di Bologna, 40138 Bologna, Italy; sara.tedeschi5@unibo.it (S.T.); eleonora.zamparini@aosp.bo.it (E.Z.); pierluigi.viale@unibo.it (P.V.); 3 Department of Medical and Surgical Sciences, Alma Mater Studiorum, University of Bologna, 40138 Bologna, Italy; 4 Department of Infectious Diseases and Tropical Medicine, Azienda Ospedaliero-Universitaria di Modena, 41124 Modena, Italy; mariannameschiari1209@gmail.com (M.M.); crimuss@unimore.it (C.M.); 5 Infectious Diseases Clinic, Santa Maria della Misericordia University Hospital of Udine, ASUFC, 33100 Udine, Italy; paola.dellasiega@asufc.sanita.fvg.it (P.D.S.); carlo.tascini@uniud.it (C.T.); 6 Infectious and Tropical Diseases Unit, Padua University Hospital, 35128 Padua, Italy; maria.mazzitelli@aopd.veneto.it (M.M.); amcattelan@libero.it (A.M.C.); 7 UOC Malattie Infettive, ASST Papa Giovanni XXIII, 24127 Bergamo, Italy; lsoavi@asst-pg23.it (L.S.); mrizzi@asst-pg23.it (M.R.); 8 UOC Malattie Infettive, Azienda Sanitaria dell’Alto Adige, 39100 Bolzano, Italy; raffaella.binazzi@sabes.it (R.B.); elkemaria.erne@sabes.it (E.M.E.)

**Keywords:** dalbavancin, population pharmacokinetics, therapeutic drug monitoring, long-term treatment, off-label use

## Abstract

A population pharmacokinetic analysis of dalbavancin was conducted in patients with different infection sites. Non-linear mixed effect modeling was used for pharmacokinetic analysis and covariate evaluation. Monte Carlo simulations assessed the probability of target attainment (PTA) of total dalbavancin concentration ≥ 8.04 mg/L over time (associated with ≥90% probability of optimal pharmacodynamic target attainment of *f*AUC_24h_/MIC > 111.1 against *S. aureus*) associated with a single or double dosage, one week apart, of 1000 or 1500 mg in patients with different classes of renal function. Sixty-nine patients with 289 concentrations were included. Most of them (53/69, 76.8%) had bone and joint infections. A two-compartment model adequately fitted dalbavancin concentration–time data. Creatinine clearance (CL_CR_) was the only covariate associated with dalbavancin clearance. Monte Carlo simulations showed that, in patients with severe renal dysfunction, the 1000 mg single or double one week apart dosage may ensure optimal PTAs of 2 and 5 weeks, respectively. In patients with preserved renal function, the 1500 mg single or double one-week apart dosage may ensure optimal PTAs of 2 and 4 to 6 weeks, respectively. Therapeutic drug monitoring should be considered mandatory for managing inter-individual variability and for supporting clinicians in long-term treatments of subacute and chronic infections.

## 1. Introduction

Multidrug-resistant (MDR) Gram-positive microorganisms such as methicillin-resistant *Staphylococcus aureus* (MRSA) or methicillin-resistant coagulase-negative staphylococci (MR-CoNS) are of great clinical concern because of the increasing emergence of antibiotic resistance [1,2], their propensity to cause catheter- and/or prosthetic-related infections and the attitude of producing biofilms within which they may be embedded [3]. 

For decades, vancomycin and teicoplanin have been the reference drugs for the treatment of MRSA- and MR-CoNS-related infections. Nowadays, reliable alternatives are daptomycin, fosfomycin, linezolid and, more recently, ceftobiprole and ceftaroline [4].

Dalbavancin is a long-acting lipoglycopeptide that is highly active in vitro against the vast majority of Gram-positive microorganisms [5,6]. It was approved in Europe in 2015 for the treatment of acute bacterial skin and skin structure infections (ABSSSI) at the dosing schedule of 1500 mg as single administration or as 1000 mg on day 1 plus 500 mg on day 8 in patients with preserved renal function or of 1000 mg as single infusion, or 750 mg on day 1 plus 375 mg on day 8 in patients with renal impairment. During the COVID-19 pandemic era, the use of this long-acting lipoglycopeptide has been progressively extended in real-life, including through the use of some off-label indications for allowing early hospital discharge of patients requiring long-term treatment (≥6 weeks), such as those with osteomyelitis, prosthetic joint infections and infective endocarditis [7]. Two population pharmacokinetic studies showed that a dosing regimen of 1500 mg one week apart may provide optimal plasma exposure against *Staphylococcus aureus* (SA) for several weeks [8,9].

Recently, we provided a proof-of-concept of the usefulness that therapeutic drug monitoring (TDM) may have in estimating the duration of dalbavancin optimal target attainment in staphylococcal osteoarticular infections [10]. Specifically, we identified that, by maintaining the total dalbavancin concentration ≥ 8.04 mg/L over time, there is a very high likelihood (≥90%) of achieving optimal pharmacodynamic target attainment (defined as *f*AUC_24h_/MIC ratio > 111.1) against SA with an MIC up to the EUCAST clinical breakpoint of susceptibility for dalbavancin (0.125 mg/L) [10].

The aim of this study was to conduct a population pharmacokinetic/pharmacodynamic analysis of dalbavancin among patients who underwent TDM during long-term treatment for different types of infection, with the aim of better defining how to properly manage dosing regimens and TDM of dalbavancin for optimal long-term treatment of subacute and/or chronic infectious diseases.

## 2. Results

### 2.1. Population Characteristics

A total of 69 patients were included in the study. The demographics and clinical characteristics of the population are summarized in Table 1. Median (minimum–maximum range) age, weight, creatinine clearance (CR_CL_) and albumin concentrations were 62 (19 to 90) years, 75 (42 to 143) kg, 93.0 (3.0 to 141.0) mL/min/1.73 m^2^ and 3.7 (2.5 to 4.6) g/dL, respectively.

Most patients (63/69, 91.3%) had single infection site. Of these, 47/63 (74.6%) had bone and joint infections, and 16/63 (34.0%) had endocarditis and/or endovascular prosthetic infections. The vast majority of patients (63/69, 91.3%) had microbiologically documented infections, with MRSA and MR-Staphylococcus epidermidis (MRSE) accounting for 74.3% (55/74) of them.

Overall, all patients received at least two doses one week apart or more at the discretion of the attending clinician. Most of them (46.4%) were treated with a two-dose regimen of dalbavancin, which, in the majority of cases, was of 1500 mg one week apart [27/32 (84.4%)]. Out of 69 patients, 17 (24.64%) were treated with a three-dose regimen of dalbavancin, whereas the other 20 patients were treated with a variable number of doses ranging from 4 to 14. The median (min–max) number of TDM per patient was 3 (1–19).

### 2.2. Population Pharmacokinetic Modeling

A total of 289 dalbavancin plasma concentrations were used to build the population pharmacokinetic model. A two-compartment pharmacokinetic model best described the dalbavancin concentration–time profiles (the OFV/BIC criteria being 2610.25/2635.66, 2406.24/2444.34 and 2403.74/2463.02 for the one-, two- and three-compartment model, respectively). The structural model building is reported in Supplementary Materials Table S1.

CL_CR_ was the only covariate significantly associated with dalbavancin clearance and included into the base model (see Supplementary Materials Table S2 for the Pearson’s correlation tests of the random effect versus covariates). After adding CL_CR_ as a power function to population CL, OFV/BIC further decreased to 2395.16/2437.49, and the inter-individual variability of dalbavancin CL decreased up to 5.32%. (see Supplementary Materials Tables S3 and S4 for covariate model building and the correlation test of individual parameter versus covariate) Theparameter estimates of both the base and the final model are shown in Table 2. The median (min–max) populations of CL, V_1_ and V_2_ were 0.043 (0.012–0.071) L/h, 6.21 (5.10–7.19) L and 9.48 (7.60–11.89) L, respectively.

**Figure 1:**
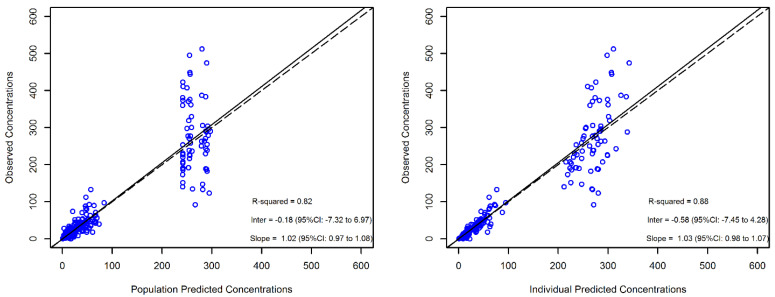
Diagnostic plot for the final population pharmacokinetic model. The observed versus population-predicted concentrations (left panel) and observed versus individual-predicted concentrations (right panel) in plasma are shown. Solid and dashed lines refer to linear regression and identity line, respectively, between the observed and the predicted concentrations.

The reliability of the final model estimates was confirmed by the low values of the relative standard error (RSE%, all less than 30%, except for the random effects on Q and V_2_) and by the high value of the coefficient of linear regression of the observed versus individual-predicted trough and peak concentrations (R^2^ = 0.88), as shown in Figure 1.

**Figure 2:**
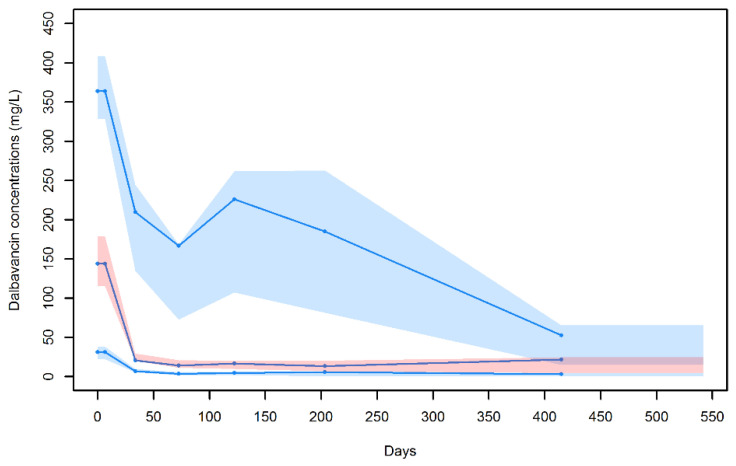
Visual predictive check for the final population pharmacokinetic model. Blue dots are the observed dalbavancin concentrations; blue lines represent the median, 10th and 90th percentiles of the observed values; and shaded areas are the prediction intervals for the median (red central area) and 10th and 90th percentiles (light blue lower and upper areas).

A good predictive performance of the model was also shown at the visual predictive check (VPC) plot (Figure 2). The symmetry test for normalized prediction distribution errors (NPDE) showed that the residuals were symmetrical around zero (*p* = 0.342) and normally distributed (*p* = 0.05 at the Shapiro–Wilk test for NPDE).

### 2.3. Monte Carlo Simulation for Estimating Pharmacodynamic Target Attainment

**Figure 3:**
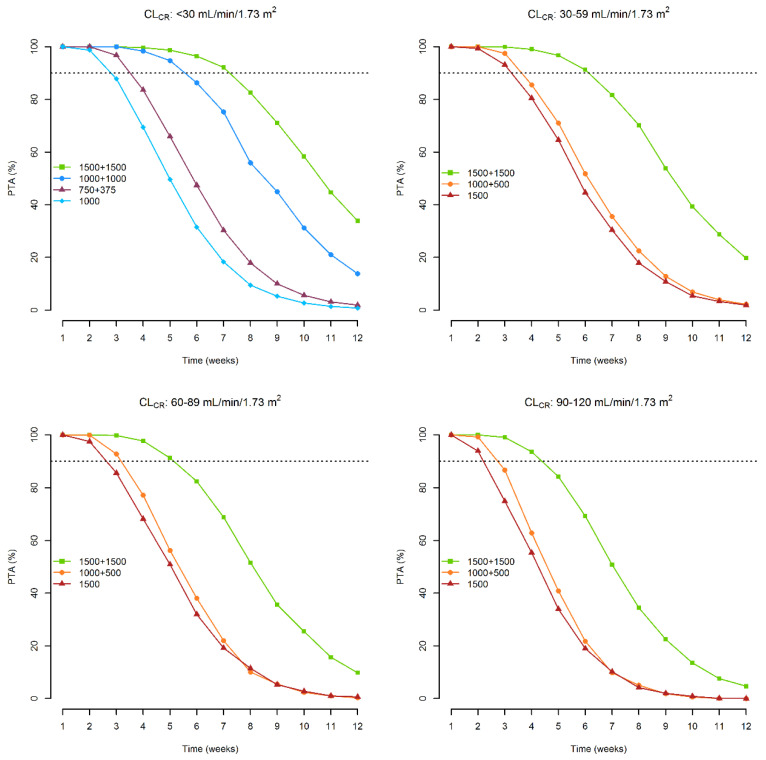
Probability of attaining a plasma concentration ≥ 8.04 mg/L over time associated with the dosages of 1500 mg on day 1, 1000 mg on day 1 + 500 mg on day 8 and 1500 mg on day 1 + 1500 mg on day 8, according to different classes of renal function. Dashed lines refer to a probability ≥ 90%.

One thousand-subject Monte Carlo simulations were performed with the approved dosing regimens (1000 mg on day 1 or 500 mg on day 1 + 375 mg on day 8 for patients with CL_CR_ < 30 mL/min/1.73 m^2^; 1500 mg on day 1 or 1000 mg on day 1 + 500 mg on day 8 for those with CL_CR_ ≥ 30 mL/min/1.73 m^2^). Two additional doubled dosing regimens were also tested: two 1000 mg doses one week apart for patients with CL_CR_ < 30 mL/min/1.73 m^2^ and two 1500 mg doses one week apart for patients with CL_CR_ ≥ 30 mL/min/1.73 m^2^. The probability of target attainment of a total plasma concentration ≥8.04 mg/L associated with these dosages is depicted in Figure 3.

With the approved dosage, optimal PTA may be granted for 2 weeks in patients with CL_CR_ of <30, 60–89, and 90–120 mL/min/1.73 m^2^, and for 3 weeks in those with CL_CR_ of 30–59 mL/min/1.73 m^2^. With the double-dosing regimen one week apart, optimal PTAs may be extended up to 4 weeks in patients with CL_CR_ of 90–120 mL/min/1.73 m^2^, 5 weeks in those with CL_CR_ of <30 and 60–89 mL/min/1.73 m^2^, and 6 weeks in those with CL_CR_ of 30–59 mL/min/1.73 m^2^.

**Figure 4:**
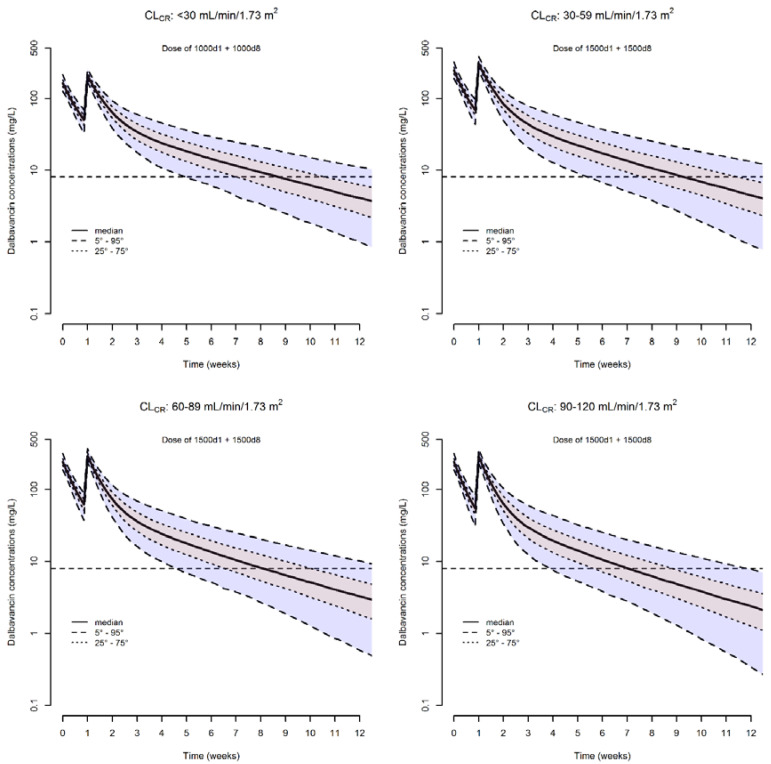
Simulated dalbavancin plasma concentration versus time profiles of a two-dosing regimen one week apart in different classes of renal function. The solid line is the simulated median concentration. The dashed lines are the 5th, 25th, 75th, and 95th percentiles of simulated concentrations. The horizontal dashed line is the threshold of concentration (8.04 mg/L) above which the desired pharmacodynamics target of *f*AUC_24h_/MIC > 111.1 is granted over time.

The median (5th–95th percentiles) simulated dalbavancin concentrations associated with the double dosages (1000 mg one week apart for patients with CL_CR_ < 30 mL/min/1.73 m^2^ and of 1500 mg one week apart in the other classes of renal function) are represented in Figure 4.

It is worth noting that despite the fact that we took into account four different classes of renal function, the distribution of plasma concentrations within each single class of CL_CR_ still remains widespread. Specifically, the range in which the 90% of concentrations are distributed is 7.9–36.0 mg/L (4.55-fold difference) at week 5 in patients with CL_CR_ < 30 mL/min/1.73 m^2^, 8.6–44.8 mg/L (5.13-fold difference) at week 6 in patients with CL_CR_ of 30–59 mL/min/1.73 m^2^, 6.9–39.0 mg/L (5.65-fold difference) at week 5 in those with CL_CR_ of 60–89 mL/min/1.73 m^2^, and 7.6–43.7 mg/L (5.75-fold difference) at week 4 in those with CL_CR_ of 90–120 mL/min/1.73 m^2^.

Based on these findings, TDM of dalbavancin should be recommended as an invaluable tool in dealing with the maintenance of optimal target attainment over time during long-term treatment. Table 3 summarizes a proposal of suggested timings for assessing the TDM of dalbavancin, in a timely and cost-effective manner, in relation to the tested dosing regimens for long-term treatment and CL_CR_ classes. The suggested timings were intended for approximately one-week in advance of the possible drop of the concentrations below the desired threshold of optimal exposure. This approach is thought to grant a very high likelihood of preventing resistance development.

## 3. Discussion

In this study, we conducted a population pharmacokinetic analysis of dalbavancin in a cohort of patients with different types of infections usually needing long-term treatment and investigated which could be the duration of optimal exposure over time according to different dosing regimens and classes of renal function.

To the best of our knowledge, this is the first population pharmacokinetic study of dalbavancin performed in real-life patients who received long-term treatment for subacute or chronic infections. Population pharmacokinetics of dalbavancin was investigated in adults in two large studies, one conducted among 532 patients (of whom, 502 had ABSSSI and 30 had bloodstream infections) [11] and the other one among 703 patients with ABSSSI [12]. Data were retrieved from registrative phase II/III clinical trials. Overall, our estimates of CL (0.043 L/h) and V (15.66 L) were very close to their findings. The two-compartment model of Buckwalter et al. [11] obtained a population CL and total V of 0.057 L/h and 15.9 L, respectively, whereas the three-compartment model of Carrothers et al. [12] obtained a population CL and total V of 0.053 L/h and 15.0 L, respectively.

Consistently with the primary renal elimination of dalbavancin and the negligible role of metabolic pathways on drug clearance [6,13], covariate analysis showed that CL_CR_ was a covariate significantly associated with dalbavancin CL, in agreement with the findings of the aforementioned population pharmacokinetic studies [11,12]. This allowed us to simulate dalbavancin concentration-versus-time profiles and to estimate the probability of attaining optimal exposure over time in four different classes of renal function.

Monte Carlo simulation with the licensed dosages showed that optimal target attainment may be granted for up to 2 weeks in all of the different classes of renal function. This is in line with proper duration of treatment for the approved indication of dalbavancin, namely ABSSIs. However, in the hypothesis of dealing with subacute or chronic infections that usually require a longer duration of treatment, these dosing schedules should not be considered as appropriate. In this regard, it is worth noting that the Monte Carlo simulation with the double dosages one week apart showed that optimal target attainment may be extended for up to 4, 5 or even 6 weeks in patients with different classes of renal function. This suggests that this double-dosing regimen could be very cost-effective in the management of subacute and/or chronic staphylococcal infections, which often need a duration of treatment of 4–6 weeks at least.

The very long elimination half-life of dalbavancin (>180 h) makes weekly administration feasible and this, coupled with a very good safety profile [5,6,14], has progressively made dalbavancin use more and more frequent in the outpatient parenteral antimicrobial treatment of several types of infections, namely bone and joint infections [15,16], infective endocarditis [17,18], endovascular prosthetic or device-related bloodstream infections [19,20], and deep sternal wound infections [21]. Interestingly, the penetration rate of dalbavancin into bone tissue was reported to be as high as 13.1% [8], whereas, to the best of our knowledge, no data about penetration into cardiac vegetations are still available to date. A recent review of real-world use of dalbavancin beyond approved indications showed that in the era of empowerment of outpatient antimicrobial treatment, the overall clinical success rate was greater than 80% in these settings [7], although data were hampered by huge differences in infection sites and by different definitions and aggregation of each single infection. It is noteworthy that, in the same review, it was highlighted that very different and heterogeneous dosing regimens were used for these purposes. Thus, important unmet needs that still need to be addressed are how to optimize the dosing schedule and how to adequately assess optimal duration for long-term treatment.

Based on our findings, we are confident that the two 1500 mg doses one week apart could be a valuable and cost-effective starting approach when dealing with long-term treatment lasting >3–4 weeks, in agreement with what was suggested by Dunne et al. [8]. However, it should not be overlooked that the Monte Carlo simulation with these dosages predicted a wide interindividual variability in drug exposure. In this regard, when looking at simulation with the two 1500 mg doses one week apart in patients without severe renal dysfunction, it is worth noting that our analysis highlighted a critical clinical issue. In fact, by stratifying the PKPD analysis in three different classes of renal function, it was shown that the duration of optimal exposure could be 2 weeks shorter in patient with normal renal function than in those with moderate renal dysfunction. Additionally, it should be considered that some patients might require longer-lasting and/or suppressive treatment, and/or that sometimes they might also have fluctuating pathophysiological conditions (namely, variable degrees of renal function over time), so that it would be very important to define properly which could be the best timing for eventually administering an additional dose.

Consequently, TDM of dalbavancin should be considered an unevaluable approach in dealing with these issues. In a previous proof-of-concept, we identified that, by maintaining the total dalbavancin concentration ≥8.04 mg/L over time, there is a very high likelihood (≥90%) of achieving optimal pharmacodynamic target attainment (defined as *f*AUC_24h_/MIC ratio > 111.1) against SA with an MIC up to the EUCAST clinical breakpoint of susceptibility for dalbavancin (0.125 mg/L) [10]. In dealing with this, our findings suggest that a very conservative and cost-effective approach to TDM of dalbavancin could be that of measuring drug exposure for the first time after a time interval ranging from 21 and 35 days, depending on the degree of the patient’s renal function. Expert interpretation of TDM results by skilled clinical pharmacologists may provide proper advice on when to reassess TDM and/or to administer an additional dose.

We acknowledge some limits of our study. The retrospective design should be recognized. A lack of testing dalbavancin susceptibility in clinical isolates precluded us from precise pharmacodynamic analysis. We recognize that the evaluation of the clinical outcome was out of scope and unfeasible, as most patients were under suppressive therapy. Finally, the inter-individual variability in pharmacokinetic parameters was appropriately estimated by the model only for CL, but not for V_1_ and V_2_. However, the overall reliability of the pharmacokinetic–pharmacodynamic model is quite high, and the predictability of its usefulness in managing long-term treatment with dalbavancin by means of TDM is a point of strength.

## 4. Materials and Methods

### 4.1. Study Design

This retrospective study was carried out between April 2021 and April 2022 among adult patients with different types of infections who underwent TDM of dalbavancin at the IRCCS, Azienda Ospedaliero Universitaria di Bologna, Bologna, Italy. The study was approved by the local Ethics Committee (registration number 897/2021/Oss/AOUBo). Signed informed consent was waived due to the retrospective and observational nature of the investigation according to hospital agreements.

Dalbavancin was started in patients with documented or suspected Gram-positive infections as a second-line treatment after failure of primary antimicrobial therapy. All the patients started with two dalbavancin doses on day 1 and on day 8. The dose amount (1000 mg or 1500 mg) was arbitrarily decided by the infectious diseases consultant at each patient center. The decision to administer any additional doses was taken in relation to the clinical evaluation of disease progression after at least 4 weeks from the administration of the second dose and it was based on the patient’s physical examination and on the trend of inflammatory biomarkers.

Therapeutic drug monitoring (TDM) of dalbavancin was applied to all included patients in order to verify the adequacy of plasma exposure during treatment and to suggest to clinicians when to apply an additional dose to prosecute treatment. TDM of dalbavancin was aimed at targeting drug concentration above 8.04 mg/L [10]. This threshold ensures the attainment of a 24 h area under the concentration–time curve for the free fraction of drug over pathogen MIC (*f*AUC_24h_/MIC) ratio of dalbavancin > 111.1, that was defined as the optimal pharmacodynamic target against *Staphylococcus aureus* [22].

Blood samples were collected at different time-points after the second dose and sent to our lab for analysis. Dalbavancin total plasma concentrations were measured by means of the liquid chromatography-tandem mass spectrometry analytic method previously described by Alebic-Kolbah T. et al. [23]. The intra- and inter-day coefficients of variation in the quality controls were 0.09% to 0.14% and 4.8% to 14.2%, respectively. The lower limit of quantification was 0.5 mg/L.

The free fraction of total dalbacancin concentration was estimated by considering a plasma protein binding of 93%, as reported in the literature [10],

Patient data were collected through a pre-specified template, which included the demographics (age, gender, weight, height), a clinical data section (type and site of infection, microbiological isolates, concomitant antibiotic treatments) and a laboratory parameter section (serum creatinine, serum albumin, C-reactive protein, procalcitonin). Creatinine clearance was estimated by means of the CKD-EPI equation [24].

### 4.2. Population Pharmacokinetic Modeling

Plasma dalbavancin concentrations were analyzed by means of nonlinear mixed-effects modeling using the stochastic approximation maximization (SAEM) algorithm implemented within the Monolix software (version 2021R1, Lixofit, Antony, France).

At a first stage, a basic model was developed, by comparing one-, two- and three-compartment models with linear elimination. All individual parameters were log-normally distributed. Exponential random effects were assumed to describe inter-individual variability. Correlations between random effects were tested in the variance–covariance matrix and implemented into the structural model accordingly. Residual variability was tested according to three different error models (constant, proportional and combined error model). Model selection was carried out according to the smaller value of the objective function value (OFV) and Bayesian information criteria (BIC), the regression coefficient of the observed versus individual predicted concentrations and the low relative standard error (RSE) of the pharmacokinetic estimates.

The following covariates were tested on the PK parameters of the base model: age, gender, weight, height, serum albumin, serum creatinine and CL_CR_. The parameter–covariate relationships were modeled as additive or proportional shifts from the reference category for binary covariates, whereas the effect of continuous covariates was modeled using a power function. A covariate was included in the model according to the results of the likelihood ratio test (LRT) and if a reduction in the BIC and a decrease in the interpatient variability of the fixed-effect parameters were obtained. Descriptions of the pharmacokinetic model building and covariate analysis are reported in the Supplementary Materials.

### 4.3. Model Evaluation

Model evaluations were based on the goodness of fit of the observed versus concentration-predicted plots, the distribution of the weighted residuals and the visual predictive check (VPC) plot. The VPC plot shows the distribution over time of the 10th, 50th and 90th percentiles of the observed concentrations in relation to the 90% prediction intervals for the same concentration percentiles, calculated from 1000 Monte Carlo samples as predicted by the model. Model performance was also assessed by means of the normalized prediction distribution error (NPDE).

### 4.4. Monte Carlo Simulation for Pharmacodynamic Target Attainment

Monte Carlo simulations from the final pharmacokinetic model were performed with Simulx 2020R1, for the recommended dalbavancin dosages of 1000 mg on day 1 and 500 mg on day 1 + 375 mg on day 8 for patients with CL_CR_ < 30 mL/min/1.73 m^2^, and of 1500 mg on day 1 and 1000 mg on day 1 + 500 mg on day 8 for those with CL_CR_ ≥ 30 mL/min/1.73 m^2^. Two additional dosages of 1000 mg one week apart for patients with CL_CR_ < 30 mL/min/1.73 m^2^ and of 1500 mg one week apart for patients with CL_CR_ ≥ 30 mL/min/1.73 m^2^ were also tested.

The probability of target attainment (PTA) of a plasma concentration ≥ 8.04 mg/L was calculated over time from week 2 to week 12. PTA ≥ 90% was defined as optimal.

## 5. Conclusions

In conclusion, our study suggests that two 1500 mg doses of dalbavancin one week apart could be a valuable starting approach when dealing with subacute and/or chronic staphylococcal infections. Assessing drug exposure by means of TDM after 3–5 weeks from starting treatment, depending on the degree of the patient’s renal function, and expert interpretation of TDM results by skilled clinical pharmacologists, may be the way forward for properly managing the duration of optimal treatment. Testing this hypothesis in a large sample size and assessing the clinical outcome would provide definitive answers on this.

## Figures and Tables

**Table 1:** Population characteristics.

Patient Demographics	
Total number of patients	69
Age (years)	62 (51–73)
Gender (male/female)	44/25
Weight (kg)	75 (62–88)
Height (cm)	170 (165–177)
Baseline laboratory parameters	
CL_CR_ (mL/min/1.73 m^2^)	93.0 (72.0–104.0)
Albumin (g/dL)	3.7 (3.3–4.0)
C-RP (mg/L)	3.21 (1.41–6.26)
Type of infections	
Prosthetic joint infection	26 (37.7)
Osteomyelitis	11 (15.9)
Endovascular prosthetic infections	9 (13.0)
Endocarditis	7 (10.1)
Spondilodiscitis	5 (7.2)
Infected pseudoarthrosis non-unions	4 (5.8)
Septic arthritis	1 (1.5)
Multiple site infections	
Endocarditis + spondilodiscitis	2 (2.9)
Endocarditis + septic arthitis	1 (1.5)
Endovascular prosthetic infection + spondilodiscitis	2 (2.9)
Endovascular prosthetic infection + osteomyelitis	1 (1.5)
Patients with identified microbiological isolates	63 (91.3)
Dalbavancin treatment	
Number of doses per patient	2 (2–4)
Number of TDM instances per patient	3 (2–5)

Data are presented as median (IQR) for continuous variables, and as number (%) for dichotomous variables. C-RP, C-reactive protein; CL_CR_, creatinine clearance; TDM, therapeutic drug monitoring.

**Table 2:** Parameter estimates of the base and final dalbavancin population pharmacokinetic models.

Parameter	Base Model Typical Value (%RSE)	Final Model Typical Value (%RSE)
Fixed-Effects		
CL (L/h)	0.041 (4.91)	0.029 (11.6)
β_CLcr-CL_	-	0.0043 (28.9)
V_1_ (L)	6.15 (4.79)	6.14 (5.26)
Q (L/h)	0.026 (17.9)	0.026 (18.1)
V_2_ (L)	10.51 (13.7)	9.52 (19.0)
Random Effects (Inter-patient %CV)		
IIV_CL_	31.76 (16.2)	26.44 (13.3)
IIV_V1_	16.10 (33.2)	16.10 (40.1)
IIV_Q_	45.06 (34.6)	50.90 (32.9)
IIV_V2_	37.19 (207)	37.15 (61.7)
Residual variability		
b (proportional)	33.92 (7.96)	33.92 (5.97)

% RSE, relative standard error of the estimate; CV, coefficient of variation; CL, total body clearance; V_1_, central volume of distribution; Q, inter-compartmental clearance; V_2_, peripheral volume of distribution; IIV, inter-individual variability (associated with CL (IIV_CL_), with V_1_ (IIV_V1_), with Q (IIV_Q_), with V_2_ (IIV_V2_)).

**Table 3:** Suggested timings (days) for assessing in a timely and cost-effective manner the TDM of dalbavancin in relation to the tested dosing regimens and CLCR classes.

Drug Dosages	Classes of CL_CR_ (mL/min/1.73 m^2^)			
	≤30	30–59	60–89	90–120
1000 d1 + 1000 d8	28 ± 3	-	-	-
1500 d1 + 1500 d8	-	35 ± 3	28 ± 3	21 ± 3

## Data Availability

The data presented in this study are available on request from the corresponding author. The data are not publicly available due to privacy concerns.

## Supplementary Materials

The following are available online at https://www.mdpi.com/article/10.3390/antibiotics11080996/s1, Table S1: Structural model building; Table S2: Pearson’s correlation test of the random effect versus covariates; Table S3: Covariate model building; Table S4: Correlation test of individual parameter versus covariates.
