# Supplementary material for: Population Pharmacokinetic and Pharmacodynamic Analysis of Dalbavancin for Long-Term Treatment of Subacute and/or Chronic Infectious Diseases: The Major Role of Therapeutic Drug Monitoring
Source: Antibiotics (Basel). 2022 Jul 24;11(8):996. doi: 10.3390/antibiotics11080996 (PMC9331863; doi:10.3390/antibiotics11080996)
Supplement: Supplementary file 1 [file antibiotics-11-00996-s001.zip › antibiotics-1771306-supplementary.pdf]

| Table S1. Structural model building | | | |
|---|---|---|---|
| | 1-compartment | 2-compartment | 3-compartment |
| Parameterization | CL, V | CL, V1, V2, Q | CL, V1, V2, V3, Q2, Q3 |
| Covariate step | Not implemented | Not implemented | Not implemented |
| OFV (-2×log-likelihood) | 2610.35 | 2406.24 | 2403.74 |
| AIC | 2622.35 | 2424.24 | 2431.74 |
| BIC | 2635.76 | 2444.34 | 2463.02 |
| BICc | 2641.49 | 2451.51 | 2474.48 |
| OFV, objective function value; AIC, Akaike information criteria, BIC(c), Bayesian information criteria (corrected) | | | |

| Table S2. Pearson's correlation test of the random effect versus covariates | | | | | | | | |
|---|---|---|---|---|---|---|---|---|
| Covariates | eta_CL | | eta_V1 | | eta_Q | | eta_V2 | |
| | Coeff | p-value | Coeff | p-value | Coeff | p-value | Coeff | p-value |
| Gender | | 3.16e-1 | | 6.77e-1 | | 3.29e-1 | | 5.36e-1 |
| Age | -0.091 | 4.55e-1 | 0.1 | 3.91e-1 | 0.18 | 1.39e-1 | 0.32 | **7.5e-3** |
| Albumin | 0.071 | 5.62e-1 | -0.24 | **4.46e-2** | -0.28 | **1.92e-2** | -0.13 | 2.76e-1 |
| BSA | 0.069 | 5.72e-1 | 0.14 | 2.5e-1 | 0.015 | 9e-1 | -0.1 | 4e-1 |
| $CL_{CR-CKDEPI}$ | 0.39 | **1.06e-3** | -0.075 | 5.39e-1 | -0.18 | 1.34e-1 | -0.12 | 3.12e-1 |
| Creatinine | -0.098 | 4.23e-1 | 0.022 | 8.61e-1 | -0.12 | 3.36e-1 | -0.16 | 1.76e-1 |
| Height | 0.14 | 2.53e-1 | -0.11 | 3.6e-1 | 0.034 | 7.81e-1 | 0.12 | 3.32e-1 |
| Weight | 0.083 | 4.95e-1 | 0.19 | 1.1e-1 | -0.0045 | 9.71e-1 | -0.026 | 8.34e-1 |
| The covariate with the highest correlation is considered for inclusion into the base structural model (forward inclusion) | | | | | | | | |

| Table S3. Covariate model building | | |
|---|---|---|
| | Base model | Covariate Model |
| Parameterization | CL, V1, V2, Q | CL, V1, V2, Q |
| Added covariate | - | $CL_{CR}$ on CL |
| OFV (-2×log-likelihood) | 2406.24 | 2395.15 |
| AIC | 2424.24 | 2415.15 |
| BIC | 2444.34 | 2437.49 |
| BICc | 2451.51 | 2444.45 |
| OFV, objective function value; AIC, Akaike information criteria, BIC(c), Bayesian information criteria (corrected) | | |

| Table S4. Correlation test of individual parameter versus covariates | | |
|---|---|---|
| | Statistics | p-value |
| Beta_CL_$CL_{CR-CKDEPI}$ | 5.09 | 3.15e-6 |
| The covariates with a p-value <0.05 should not be removed from the model (backward elimination) | | |

**Description of the pharmacokinetic model building and covariate analysis**

Table S1 reports the values of the OFV, AIC, BIC and BICc used to select the base population pharmacokinetic model. The model with the lowest values (i.e. the 2-compartment model), was finally selected. Table S2 reports the Pearson's correlation test of the random effect of the PK parameter of the base model versus all the tested covariates. As the lowest p-values was between eta_CL and $CL_{CR}$. $CL_{CR}$ was added as a covariate on dalbavancin CL. Table S3 shows the values of the OFV, AIC, BIC and BICc after including $CL_{CR}$ on CL. As all these values decreased compared to those of the base model, $CL_{CR}$ was definitely included into the final population pharmacokinetic model. Table S4 shows the result of the correlation test of individual parameter versus covariates, and confirms that $CL_{CR}$ should not be removed from the final model.
